# Synthesis of Fluorescent Carbon Dots as Selective and Sensitive Probes for Cupric Ions and Cell Imaging

**DOI:** 10.3390/molecules24091785

**Published:** 2019-05-08

**Authors:** Shu-Wei Huang, Yu-Feng Lin, Yu-Xuan Li, Cho-Chun Hu, Tai-Chia Chiu

**Affiliations:** 1 Department of Applied Science, National Taitung University, Taitung 95092, Taiwan; shuwei0615@gmail.com (S.-W.H.); danny861106@gmail.com (Y.-X.L.); cchu@nttu.edu.tw (C.-C.H.); 2 Department of Biomedical Engineering and Environmental Science, National Tsing Hua University, Hsinchu 30013, Taiwan; assassin52852@gmail.com

**Keywords:** cell imaging, cupric ion, cysteine, fluorescent carbon nanodots, poly(vinylpyrrolidone)

## Abstract

A novel sensing system has been designed for the detection of cupric ions. It is based on the quenched fluorescence signal of carbon dots (CDs), which were carbonized from poly(vinylpyrrolidone) (PVP) and L-Cysteine (CYS). Cupric ions interact with the nitrogen and sulfur atoms on surface of the CDs to form an absorbed complex; this results in strong quenching of the fluorescence of the CDs via a fast metal-to-ligand binding affinity. The synthesized water-soluble CDs also exhibited a quantum yield of 7.6%, with favorable photoluminescent properties and good photostability. The fluorescence intensity of the CDs was very stable in high ionic strength (up to 1.0 M NaCl) and over a wide range of pH levels (2.0–12.0). This facile method can therefore develop a sensor that offers reliable, fast, and selective detection of cupric ions with a detection limit down to 0.15 μM and a linear range from 0.5 to 7.0 μM (*R*^2^ = 0.980). The CDs were used for cell imaging, observed that they were low toxicity to Tramp C1 cells and exhibited blue and green and red fluorescence under a fluorescence microscope. In summary, the CDs exhibited excellent fluorescence properties, and could be applied to the selective and sensitive detection of cupric ion and multicolor cell imaging.

## 1. Introduction

Heavy metal ion contamination is an important public health concern [1,2,3]. Cupric ions (Cu^2+^) are a key trace element in the human body and they play a critical role in many biological processes, such as in metabolism regulation, oxidation, and protein activation [4,5]. As such, a small intake of Cu^2+^ can be beneficial for people, but taking in too much can lead to toxicity-related problems, as it may harm vital organs and potentially induce a variety of serious diseases [6,7,8]. It is therefore important that sensitive and selective Cu^2+^ detection methods are developed. Researchers have developed a number of such methods, including atomic absorption spectrometry [9], inductively coupled plasma mass spectrometry [10], and electrochemical methods [11]. Although these methods are highly sensitive, they utilize expensive instruments, have high operating costs, require complicated sample preparations, and have tedious operating procedures; these methods are therefore unsuitable for monitoring purposes in the field. Simple, sensitive, selective, and low-cost methods for measuring Cu^2+^ ions in the field therefore need to be developed. Development of novel sensors is of great interest to chemists because of their low-cost, simplicity, high selectivity and sensitivity. Various organic fluorophore-based sensors have been reported for the determination of Cu^2+^ with relatively high sensitivity [12,13], but traditional organic dyes often lack photostability, produce narrow excitation and broad emission bands, which limits their performance in practical applications. Recently, carbon dots (CDs) provides an alternative way for the detection of Cu^2+^. Xu et al. [14] discovered CDs in 2004, and scientists have since continuously explored new ways in which to prepare them. Various approaches for synthesizing CDs have been reported upon, including ultrasonic synthesis, chemical oxidation, and electrochemical synthesis, as well as microwave, pyrolysis, and hydrothermal carbonization [15,16,17,18]. CDs have attracted a lot of attention due to their chemical inertness, high stability, resistance to photobleaching, bright fluorescence, low toxicity, excellent aqueous solubility, and biocompatibility; these characteristics have caused them to be applied to many fields, including sensing, bioimaging, optoelectronic devices, and photocatalysis [19,20,21,22]. These characteristics also make CDs more suitable than other fluorescent materials for use as probes in the detection of Cu^2+^.

Many high-quality Cu^2+^ sensors that utilize modified CDs have been reported. A ratiometric fluorescence nanosensor that could selectively detect Cu^2+^ was developed by Wang et al. [23]; their sensor covalently connected carboxyl-modified cadmium telluride quantum dots to amino-functionalized CDs; the limit of detection (LOD) for Cu^2+^ was down to 0.36 nM. A highly selective phosphorous and nitrogen co-doped CDs were prepared using a low-temperature carbonization method as demonstrated by Omer [24]. The CDs successfully detected Cu^2+^ with the limit of detection as low as 1.5 nM. Bhamore et al. [25] demonstrated the fabrication of fluorescent CDs by using *Acacia concinna* seeds as precursors and the CDs was developed for sensing Cu^2+^. The detection limit was down to 4.3 nM.

The good water solubility, photostability, and biocompatibility makes CDs favorable for cell imaging as optical probes. Konar et al. [26] reported the preparation of multicolor CDs from tartaric acid and urea, and using them to detect the cysteamine and cell image. One-pot sonochemical-assisted synthesis of nitrogen doped CDs derived from crab shell was demonstrated by Dehvari et al. [27]. The CDs had been used for the multicolor image of lung and HeLa cells. Pathak et al. [28] developed nitrogen and sulfur co-doped CDs and showed excellent multicolor imaging for various pathogenic bacteria and human buccal epithelial cells. The only disadvantage of CDs is due to their low fluorescence quantum yield (QY) compared to quantum dots containing cadmium or other heavy metals. Therefore, the fluorescence intensity of CDs can be enhanced through the passivation of the surface through conjugation with suitable electron donor groups or by doping them with various electron rich heteroatoms. These strategies can considerably improve the quantum yield of CDs.

In this study, a one-pot synthesis assay for preparing nitrogen- and sulfur-co-doped fluorescent CDs from poly(vinylpyrrolidone) (PVP) and L-Cysteine (CYS) is proposed; a hydrothermal method is used for their preparation. The CDs were found to exhibit strong blue photoluminescence and possess both good photostability and high solubility. This assay was a simple, label-free, selective and sensitive sensing method for detecting Cu^2+^ in environmental water and multicolor cell imaging.

## 2. Materials and Methods

### 2.1. Chemicals and Reagents

PVP, CYS, H_3_PO_4_, NaH_2_PO_4_, Na_2_HPO_4_, Na_3_PO_4_, quinine sulfate, KCl, NaCl, CuCl_2_, HgCl_2_, PbCl_2_, ZnCl_2_, MnCl_2_, CoCl_2_, MgCl_2_, FeCl_3_, CaCO_3_, HCl, H_2_SO_4_, isopropanol and 3-(4,5-dimethylthiazol-2-yl)-2,5-diphenyltetrazolium bromide (MTT) were purchased from Sigma-Aldrich (St. Louis, MO, USA). Tris(hydroxymethyl)aminomethane (Tris) was purchased from J. T. Baker (Phillipsburg, NJ, USA). All chemical reagents were the analytical reagent grade and used without further purification. Deionized (DI) water was collected from a Barnstead Nanopure Ultrafiltration Unit (Boston, MA, USA).

### 2.2. Instruments and Characterization

UV–Vis absorption spectra were recorded by a Lambda EZ210 spectrophotometer (Perkin Elmer, Waltham, MA, USA). Fluorescence spectra were recorded by an F-7000 fluorescence spectrophotometer (Hitachi, Tokyo, Japan). Transmission electron microscopy (TEM) measurements were performed using a JEM-2100 transmission electron microscope (JEOL, Tokyo, Japan). X-ray photoelectron spectroscopy (XPS) analyses were performed using an ESCALAB 250 X-ray photoelectron spectrometer (Thermo Fisher Scientific, Waltham, MA, USA). IR spectra were measured using a Fourier transform infrared (FTIR) spectrometer (Perkin Elmer, Waltham, MA, USA).

### 2.3. Synthesis of the CDs

A one-pot hydrothermal-assisted pyrolysis approach was used to obtain the CDs. Typically, PVP (0.5 g) and CYS (0.5 g) was dissolved in 15 mL DI water, and then the mixture was transferred into a 50-mL Teflon-lined stainless-steel autoclave and heated at a constant temperature of 180 °C for 12 h. After cooling to room temperature, the obtained yellow-colored solution was centrifuged at 12,000 rpm for 10 min. Then, the collected supernatant was filtered through a 0.22-μm membrane (Millipore, USA) to remove large particles. The CDs was then dialyzed using DI (2.5 L) water through a dialysis membrane (MWCO 3500 Da) for 6 h to remove tiny fragments and any remaining salts. The resulting CDs exhibited blue luminescence under a 365-nm UV light. 

### 2.4. Quantum Yield Calculation

The relative quantum yield of the CDs was calculated using a quinine sulfate solution in 0.1 M sulfuric acid (*Q*_R_ = 0.54) as a reference. The following equation was used to calculate CDs QY:

Q_CDs_ = Q_R_ × (I_CDs_/I_R_) × (A_R_/A_CDs_) × (*n*^2^_CDs_/*n*^2^_R_) (1)

where *Q*_R_ is the QY of the reference compound, *n* is the refractive index of the solvent (1.33 for water), *I* is the integrated fluorescence intensity, and *A* is the absorbance at the excitation wavelength. For the real absorption effects to be minimized, the absorbance of the CDs was maintained under 0.05 at the excitation wavelength of 340 nm.

### 2.5. Cu^2+^ Fluorescence Assay

Cu^2+^ sensing was performed at room temperature using Tris–HCl (10 mM, pH 6.0) buffers. A desired amount of Cu^2+^ (final concentration of 0–500 μM) was added to a 1.0 mL Tris–HCl buffer containing the CDs. The fluorescence emission spectra were measured using an excitation wavelength of 355 nm after a reaction had been carried out for 1 h. 

### 2.6. Analysis of a real sample

The water sample was collected from Jinsin Lake in the National Taitung University (Taitung, Taiwan). The sample was centrifuged at 12,000 rpm for 10 min and filtered through a 0.22-μm membrane prior to detection being performed. An aliquot (100 μL) of the water sample was spiked with a standard Cu^2+^ solution (100 μL, final concentrations were 1.0 and 5.0 μM, respectively). The samples containing Cu^2+^ were diluted to 1.0 mL with Tris–HCl buffer containing the CDs and then analyzed using the developed sensing approach. 

### 2.7. Cell Viability Assay

The MTT assay was used to analyze the cytotoxicity of CDs. In brief, Tramp C1 cells (CCL-2730, epithelial of prostate cancer cell from transgenic mouse, ATCC, Manassas, VA, United States) were seeded in 96-well plates at a density of 5 × 10^3^ cells/well and cultured for 12 h at 37 °C in a humidified incubator. Different concentrations of CDs from 0.3125 mg/mL up to 10.0 mg/mL were incubated with the cells in the complete medium for 24 h. Then, the culture media were removed and the MTT solution (100 μL, 0.5 mg/mL in PBS) was added to each well, followed by incubation at 37 °C for 30 min. The supernatant was removed, and 100 μL isopropanol was added to dissolve the formed formazan. After shaking the plates, absorbance values of the wells were obtained using the microplate reader at 490 nm. The cell viability was calculated using the following equation:

Cell viability (%) = (A_CDs_/A_cell_) × 100%, (2)

where A_CDs_ is the absorbance of the experimental group (i.e., the cells were treated with CDs) and A_cell_ is the absorbance of the control group (i.e., the cells without any treatment).

### 2.8. Cell Image Studies

Tramp C1 cells (1.5 × 10^3^) were seeded onto 10 mm round glass slide and cultured for 12 h at 37 °C in a humidified incubator. The CDs at the concentration of 2.5 mg/mL were added to the cells and were further incubated for 6 h. After incubation with CDs, cells were fixed with 4% paraformaldehyde for 30 min and were mounted on the quartz slide with the ProLong Gold Antifade mounting medium overnight. Finally, the CDs staining cells were analyzed using an inverted fluorescence microscope (IX-71, Olympus, Center Valley) under 40× oil objective.

## 3. Results

### 3.1. Characterization of the CDs

**Scheme 1:**
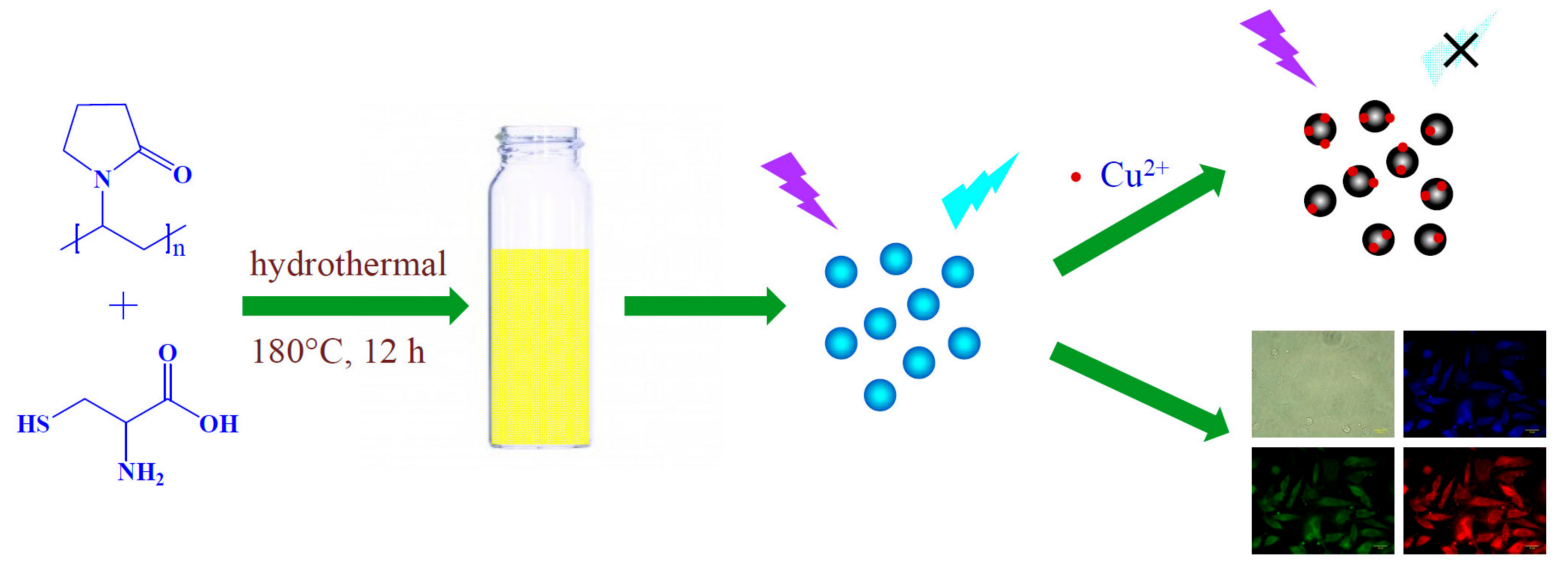
Schematic illustration of the synthesis of carbon nanodots via hydrothermal treatment for Cu^2+^ detection and cell imaging.

It has been reported that doped N and S cause CDs to possess heteroatoms in their carbon frameworks, which influences their photoluminescence [29,30,31,32]. For this reason, a freshly prepared colorless PVP and CYS solution was sealed in a Teflon-lined stainless-steel autoclave that was heated at a constant temperature. The CDs formation process included the condensation of both PVP and CYS, and the carbonization process is shown in Scheme 1. The water-soluble fluorescent N/S-CDs were obtained by a one-pot hydrothermal method in which PVP and CYS were used as the carbon, nitrogen, and sulfur sources. The results for the optimization of synthetic condition of CDs for the determination Cu^2+^ were listed in Table S1. For the purpose of getting higher sensitivity, the CDs were prepared through a hydrothermal process of 0.5 g PVP and 0.5 g CYS at 180 °C for 12 h. Under these conditions, the good batch-to-batch reproducibility was achieved (Figure S1).

**Figure 1:**
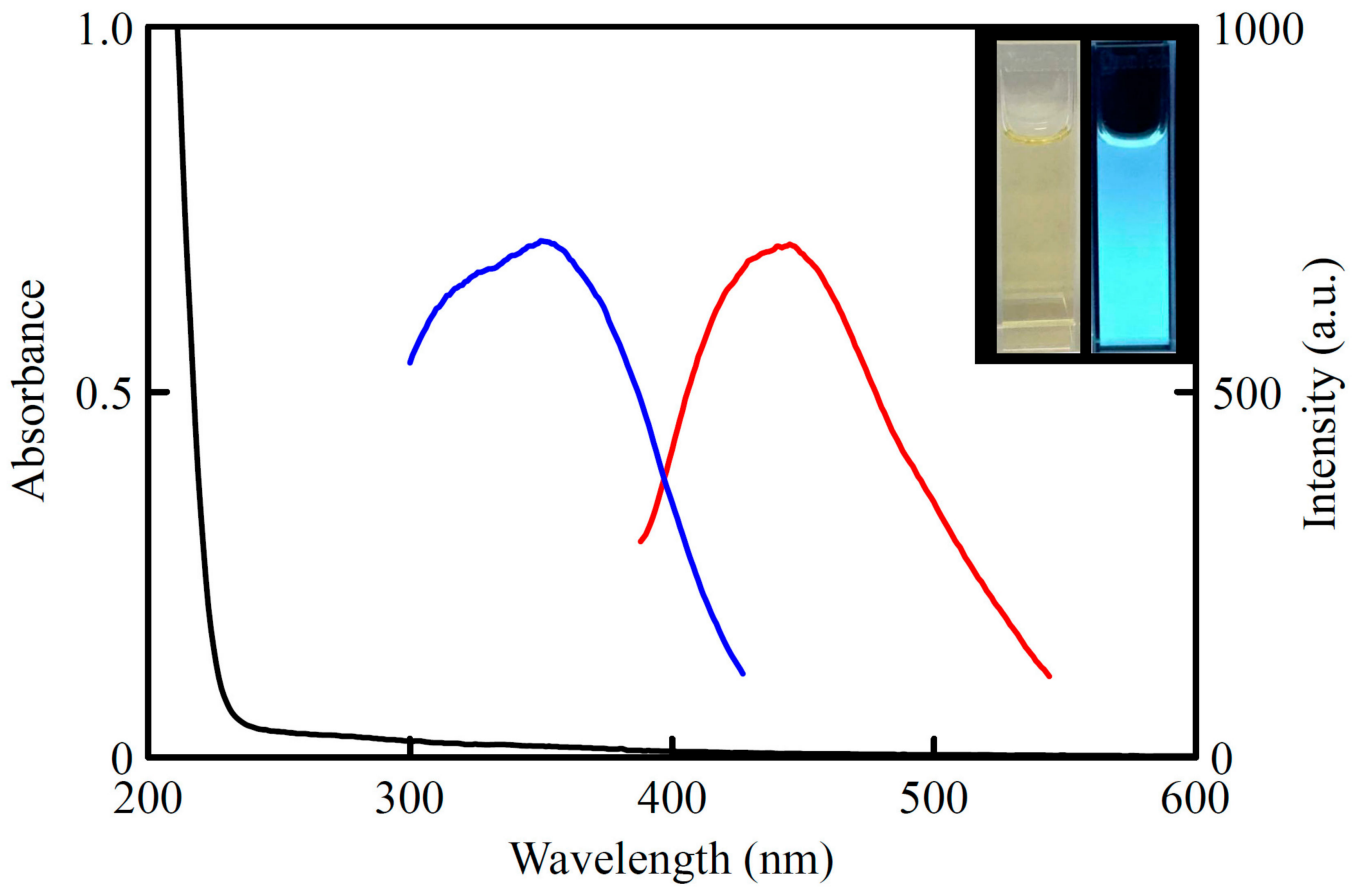
UV–Vis absorption spectrum (black) and fluorescence spectra (excitation spectrum: blue; emission spectrum: red) of the carbon dots (CDs). Inset: Samples excited by visible light and a 365-nm UV lamp.

The UV–Vis absorption, excitation, and emission spectra of the CD solution are shown in Figure 1. The CDs were observed to exhibit a very broad absorption band from 235 to 400 nm. This band was consistent with that of previously reported functionalized CDs [33]. The CDs were found to be well dispersed; they exhibited a yellowish and transparent solution under visible light, and they exhibited intense blue luminescence under UV irradiation (Figure 1, inset). The as-prepared CDs were able to fluoresce over excitation wavelengths from 300 to 400 nm, and the maximum fluorescence emission occurred at an excitation wavelength of 355 nm. Using quinine sulfate as a standard, we calculated that the fluorescence QY of the CDs was about 7.6%. As shown in Table S2, most hydrothermal methods had higher QY (> 30%). Additionally, the maximum emission wavelength became red-shifted as the excitation wavelength increased (Figure S2). The excitation-dependent emission behavior of the CDs observed in this study has also been reported in previous studies [33].

The morphology and particle size of the CDs were examined by TEM (Figure S3); it was found that the CDs had both good monodispersity and size distribution, with particle sizes of 14–26 nm; the average diameter of the particles was 21 nm (RSD = 16%).

**Figure 2:**
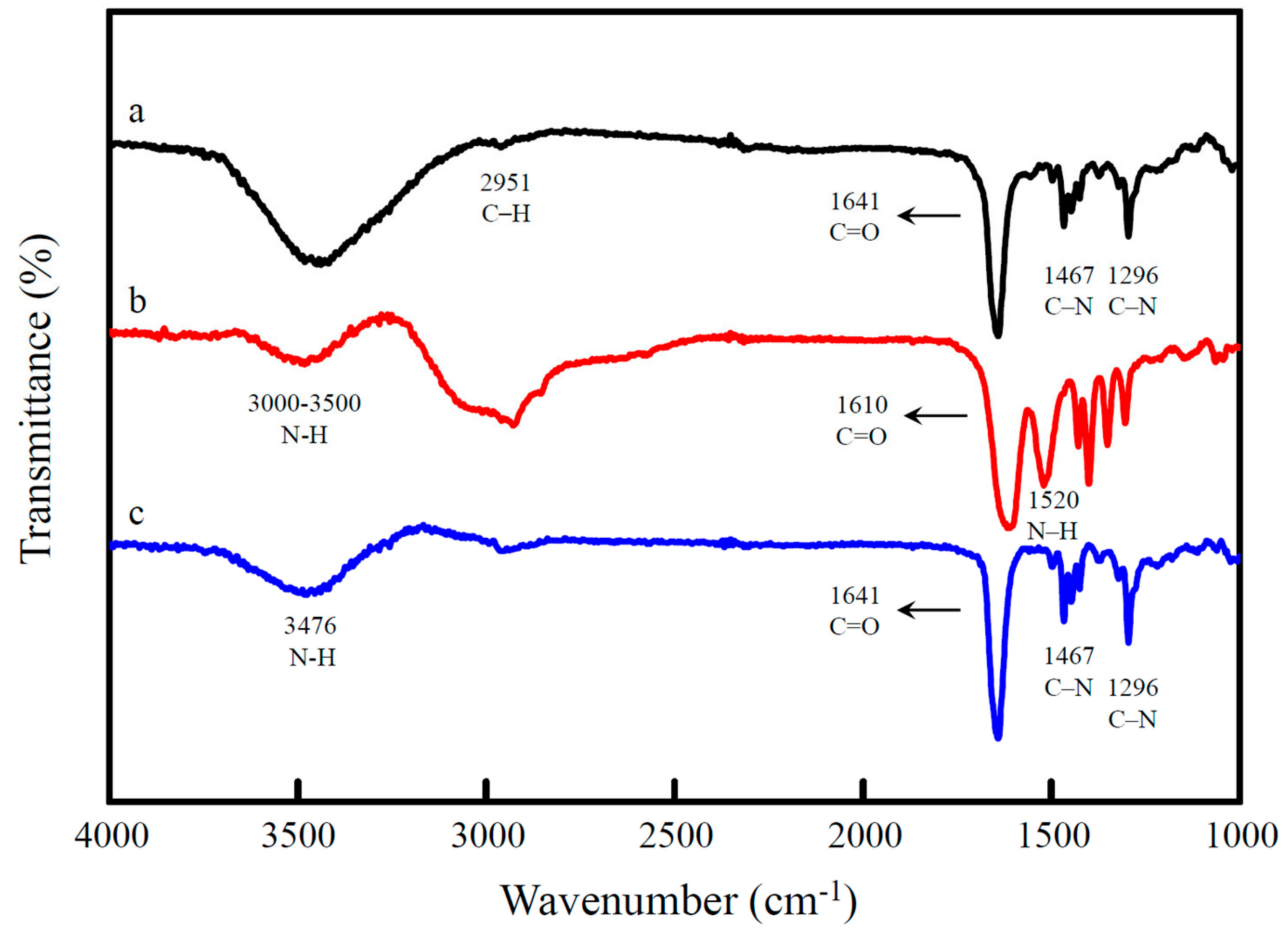
FTIR spectra of (**a**) poly(vinylpyrrolidone) (PVP), (**b**) L-Cysteine (CYS), and (**c**) the CDs.

FTIR and XPS analyses were performed to study the functional groups and chemical compositions of the as-prepared CDs. Figure 2 shows the FTIR spectra of PVP, CYS, and the CDs. A prominent peak at around 3450 cm^−1^ is present in all three samples; this can therefore be ascribed to the OH stretching vibration modes of the surface-absorbed water molecules [33]. In the spectrum obtained for PVP, the peak at 2951 cm^−1^ can be attributed to an asymmetric C–H stretching vibration. The peak located at 1641 cm^−1^ for PVP was determined to be related to the C=O group of *N*-vinyl pyrrolidone, and the C–N bending and stretching vibrations were believed to be the causes for the peaks 1467 and 1296 cm^−1^, respectively [34]. The FTIR spectrum of CYS is shown in Figure 2b. The characteristic bands at 1610 and 1401 cm^−1^ are due to the asymmetric and symmetric stretching vibrations, respectively, of the carbonyl group [35]. The band at 1520 cm^−1^ corresponds to a N–H band, and a very broad band of NH_3_^+^ stretching is observed for 3000–3500 cm^−1^. The weak peak at around 2560 cm^−1^ represents the S–H group of CYS [35]. There is no characteristic CYS bands observed in the FTIR spectra of CDs, may be due to that CYS has been bonded in the core of CDs not on the surface of CDs. The FTIR absorption bands at 3476, 1641, 1467, and 1296 cm^−1^ (Figure 2c) suggest that there are many amine, hydroxyl, and carboxyl groups on the surface of the as-prepared CDs, which is why the CDs exhibited excellent water solubility. 

**Figure 3:**
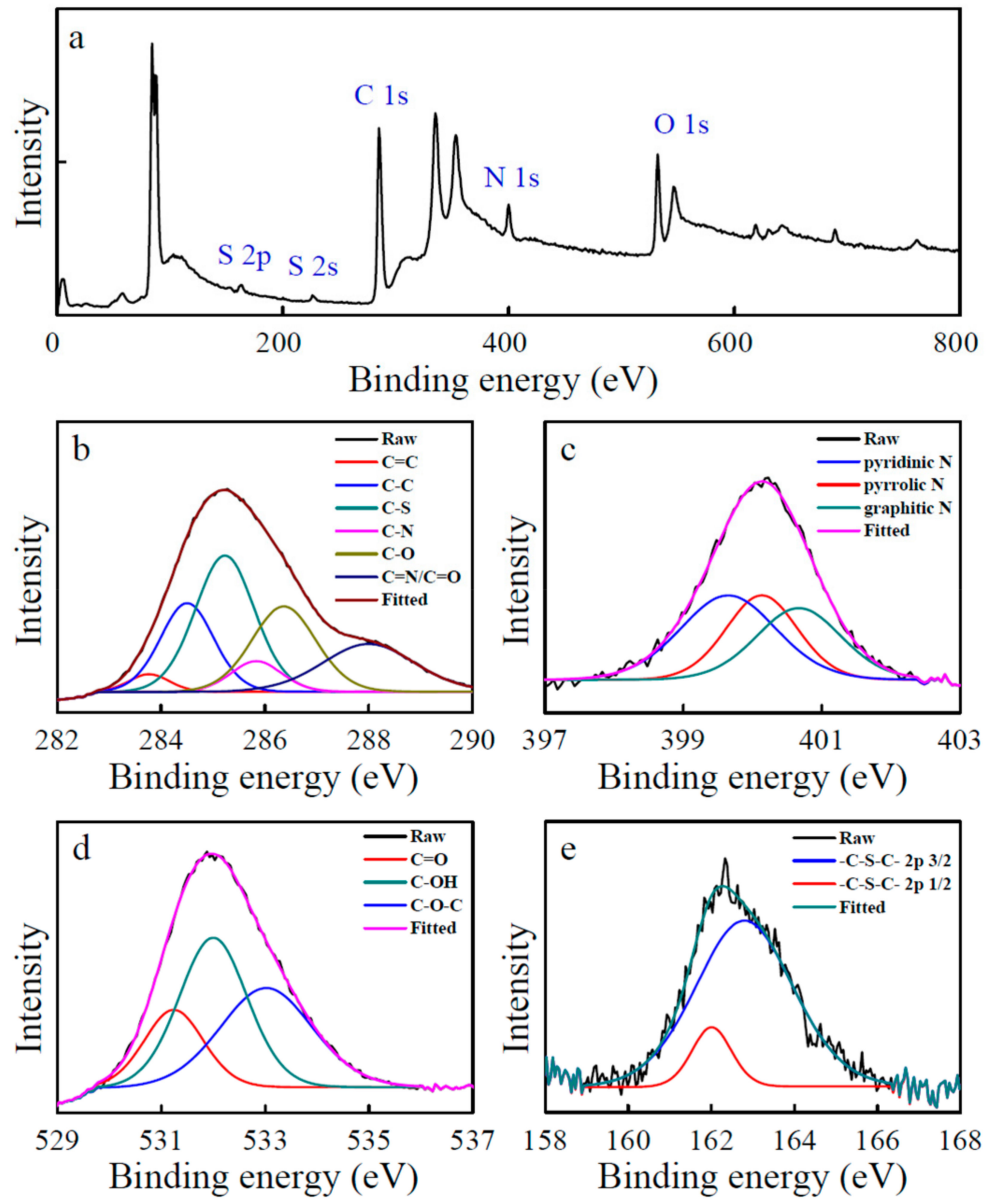
XPS spectra of the CDs: (**a**) Full-scan XPS spectrum. High-resolution XPS spectra of (**b**) C 1s, (**c**) N 1s, (**d**) O 1s, and (**e**) S 2p.

The full-scan XPS spectrum of the CDs is shown in Figure 3a; excluding the three peaks that can be seen at around 532, 400, and 285 eV, which corresponded to O 1s, N 1s, and C 1s, respectively, the two peaks at 226 and 163 eV are probably due to S 1s and S 2p, respectively. These results indicate that the CDs were mainly composed of four elements: C, N, O, and S. The high-resolution C 1s spectrum (Figure 3b) consisted of six component peaks, which we attributed to C=C (283.8 eV), C–C (284.5 eV), C–S (285.2 eV), C–N (285.8 eV), C–O (286.4 eV), and C=N/C=O (288.0 eV) [33]. The high-resolution N 1s spectrum of the CDs (Figure 3c) shows the presence of pyridinic N (399.6 eV), pyrrolic N (400.0 eV), and graphitic N (400.7 eV) [36]. The peaks in the high-resolution spectrum of O 1s (Figure 3d) indicate that there were three characteristic oxygen states: C=O (531.2 eV), C–OH (531.9 eV), and C–O (533.0 eV) [37]. The S 2p spectrum exhibited two peaks at 162.0 and 162.8 eV (Figure 3e), which correspond to –C–S–C– 2p 3/2 and–C–S–C– 2p 1/2 bonds, respectively [36]. The FTIR and XPS spectra therefore indicated that the CDs consisted of functional groups with oxygen, nitrogen, and sulfur elements, and these endowed the CDs with excellent dispersibility in aqueous solutions without any further modifications being required.

### 3.2. Fluorescence Stability of the CDs

To verify the stability of the CDs, we measured how representative pH levels, ionic strengths, illuminations over long periods of time, and storage times affected their fluorescence intensity. At pH values from 2.0 to 12.0, the fluorescence intensity of the CDs was very stable (Figure S4a). As Cu^2+^ will form complexes with hydroxide at higher pH levels, pH 6.0 was selected as being an optimum pH level. The fluorescence intensity also remained constant at NaCl concentrations to 1.0 M at pH 6.0 (Figure S4b). It was seen that the fluorescence intensity only decreased by about 3% in 1 h under continuous UV light (365 nm) illumination (Figure S4c). The CDs were also found to exhibit good photostability when stored at 4 °C in the dark for about 4 months (Figure S4d). These results suggest that the CDs have stable fluorescent properties.

### 3.3. Detection of Cu^2+^

**Figure 4:**
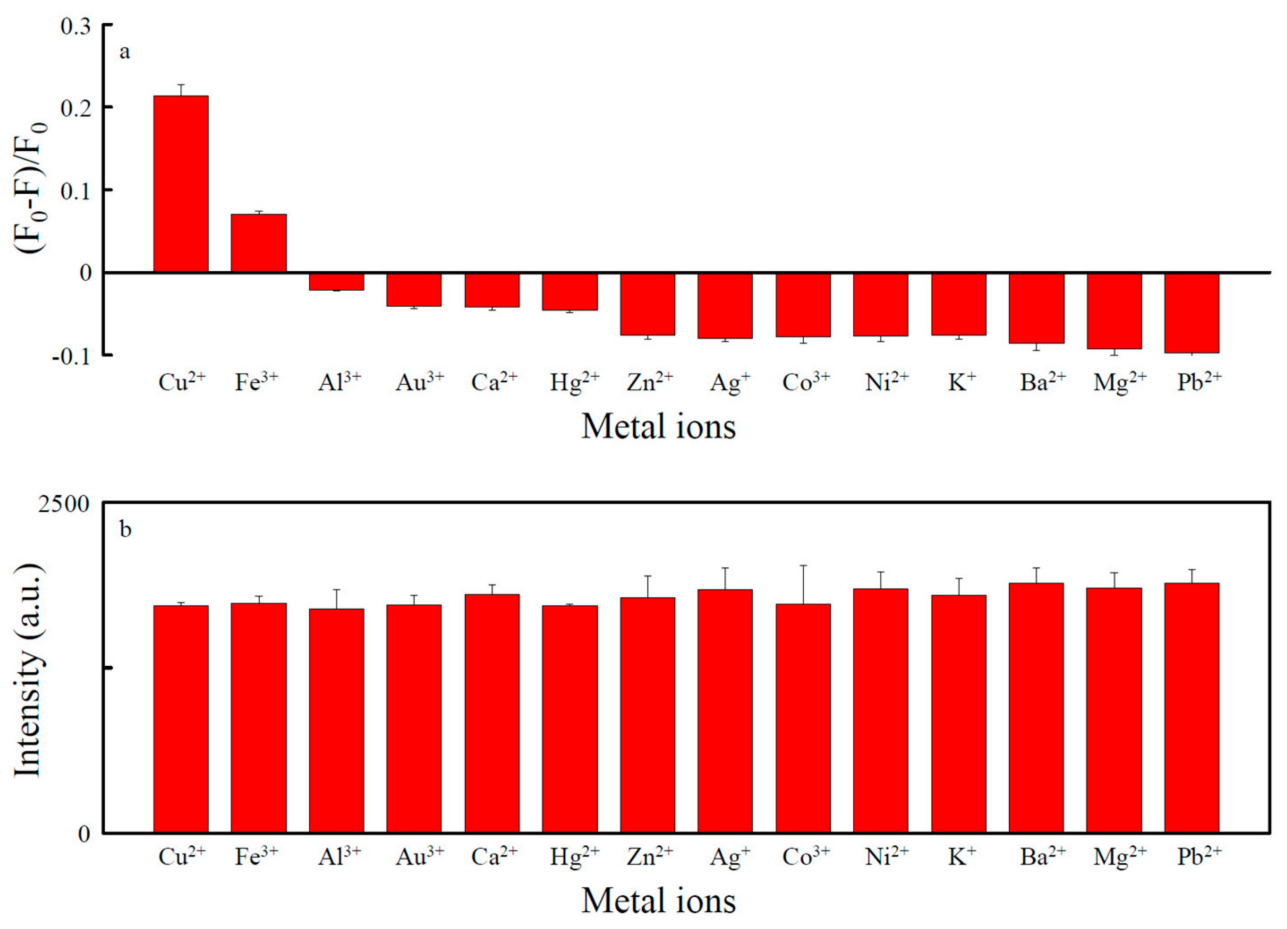
(**a**) Selectivity of the CDs as a probe for metal ions (10.0 μM) in a Tris–HCl buffer (1.0 mM, pH 6.0); (**b**) response profiles of the CDs by Cu^2+^ in the presence of different metal ions in a Tris–HCl buffer (1.0 mM, pH 6.0).

As discussed in the introduction, the N,S-based functional group on the surfaces of the CDs was expected to make the nanosensor suitable for the detection of Cu^2+^, because of the strong interactions that would occur between the functional groups and these metal ions [38]. In the present work, the potential of using these CDs as probes for metal ions (10 μM) was explored in detail by measuring the fluorescence changes of the developed CDs in a Tris–HCl buffer (1.0 mM, pH 6.0). The results are as shown in Figure 4a, where *F*_0_ and *F* are the fluorescence intensities of the probes at 455 nm in the absence and presence of the Cu^2+^ ions, respectively. Notably, of the 14 metal ions tested, Cu^2+^ was found to have the highest selectivity toward the fluorescence quenching of the CDs. The mechanism involved in the process can be attributed to the particularly high thermodynamic affinity of Cu^2+^ for the N,S-chelate groups on the surfaces of the CDs and the fast metal-to-ligand binding kinetics [39]. The possible interference of coexisting cations in the Cu^2+^ detection process was also investigated (Figure 4b), and the response of the CDs to Cu^2+^ was almost unchanged both before and after the addition of interfering ions. These results indicate that the fluorescent probe we have proposed exhibits high selectivity when detecting Cu^2+^.

The linear range, sensitivity, and LOD of the proposed sensing system for Cu^2+^ was investigated under optimum conditions. Figure S5 shows the fluorescence spectra of the CDs for various Cu^2+^ concentrations. The fluorescence intensity decreased linearly as the concentration of Cu^2+^ increased from 0 to 500 μM; this indicates that Cu^2+^ bonded effectively with the N,S-based functional groups on the surfaces of the CDs. The plot of (*F*_0_ − *F*)/*F*_0_ as a function of the Cu^2+^ concentration in the range of 0.5–7.0 μM is shown in the inset of Figure S5. The correlation coefficient, *R*^2^, is 0.980, which indicates that there is good linearity between these two parameters. Under this optimum condition, the LOD was calculated as being 0.15 μM (at a S/N of 3), which is comparable to other reported probes in Table S2. Most hydrothermal approaches exhibited high quantum yield and lower LODs, but the solvothermal approach showed the highest quantum yield (78.6%). The lowest LOD for the determination of Cu^2+^ was achieved by using N-doped quantum dots prepared by the pyrolysis of ammonium citrate.

### 3.4. Detection of Cu^2+^ in a Water Sample

The feasibility of using the CDs for detecting Cu^2+^ in water samples was evaluated. The measurements were done after adding a known amount of Cu^2+^ to water samples from an environmental water source. The Cu^2+^ in the lake water was detected according to the procedure explained in the experimental section. The addition of Cu^2+^ to the water samples led to a decrease in the fluorescence intensity of the CDs. To evaluate the reliability of the CDs for detecting Cu^2+^ in environmental water, spiked-recovery experiments were carried out with the lake water, and the recoveries were 91.4–102.0%, as shown in Table 1. These results indicated that the CDs were capable of detecting Cu^2+^ in environmental waters samples.

### 3.5. Cytotoxicity and Cell Imaging

**Figure 5:**
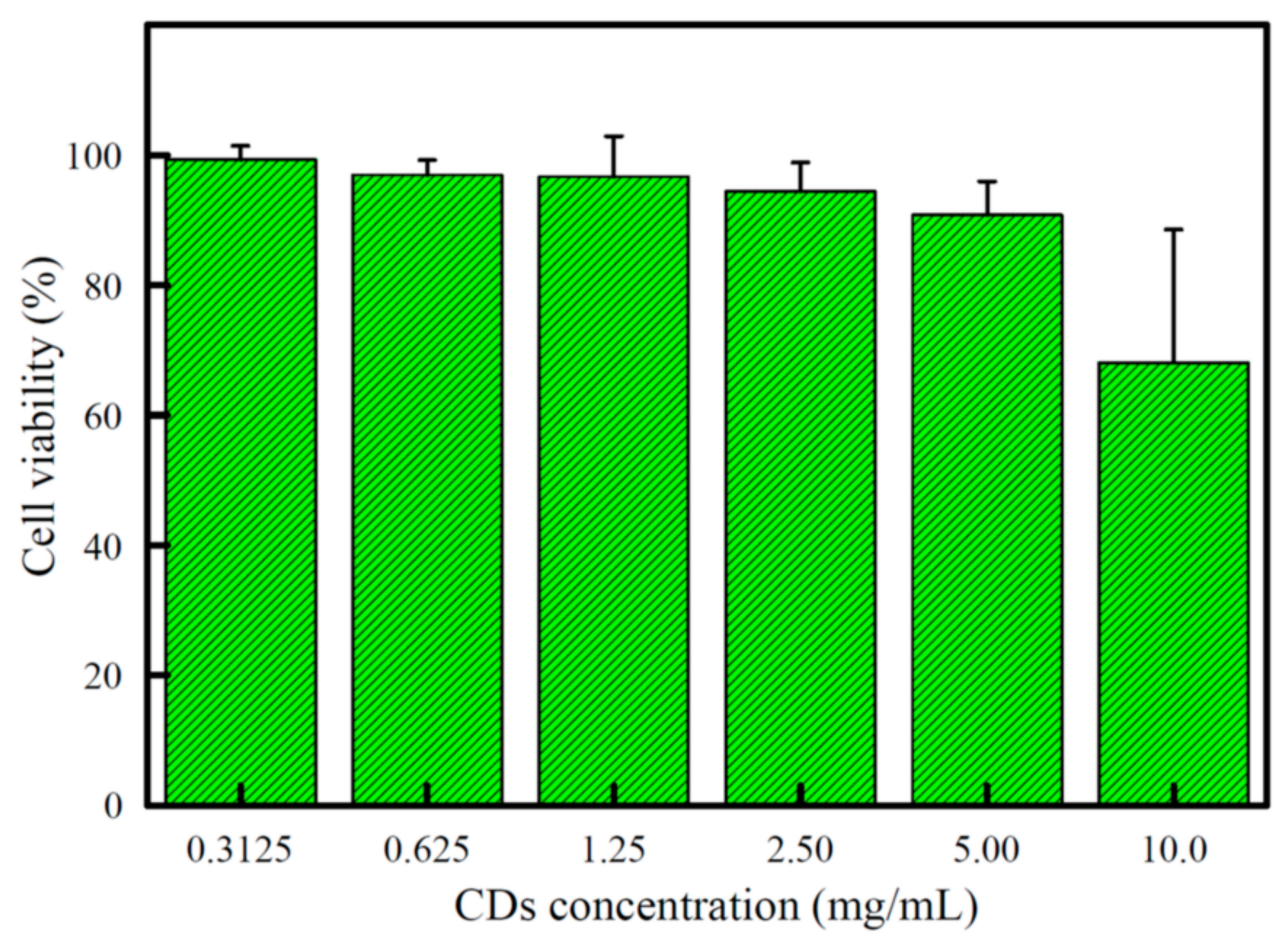
Cell viabili ty of Tramp C1 cells after incubation with different concentrations of CDs for 24 h.

**Figure 6:**
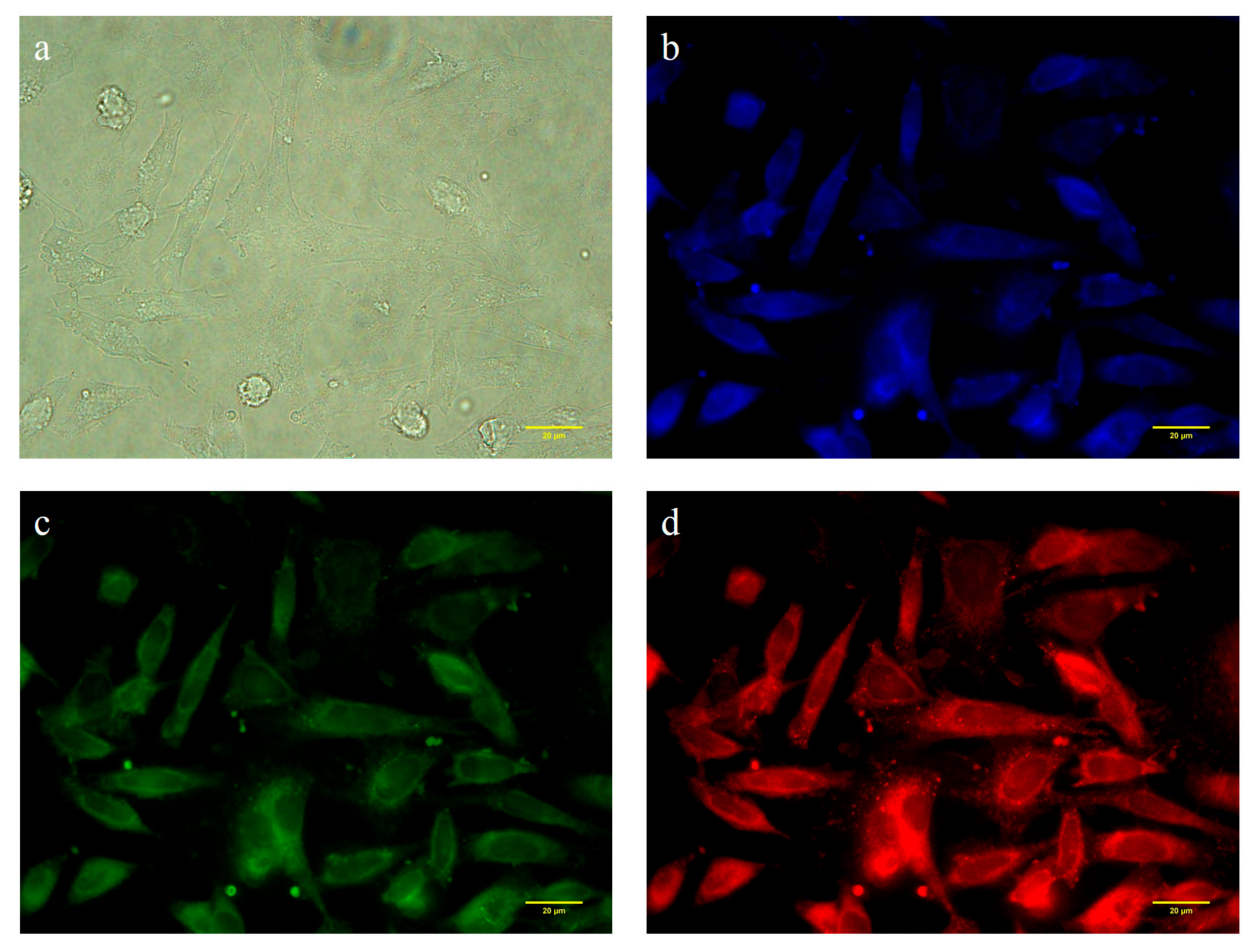
Fluorescence image of Tramp C1 cells staining with CDs: (**a**) The bright-field images and corresponding images under different fluorescence channel; (**b**) DAPI channel: Ex: 350–400 nm, Em: 420–470 nm, DC: Without setting; (**c**) WB channel: Ex: 460–490 nm, Em: 520 nm long pass, DC: 500 nm and (**d**) WG channel: Ex: 510–550nm, Em: 590 nm long pass, DC: 570 nm. Scale bar = 20 μm.

To explore the potential application of CDs in cell-labeling and imaging, Tramp C1 cells were used to evaluate the cytotoxicity of CDs by the MTT assay. CDs with different concentrations were incubated with cytotoxicity of Tramp C1 cells for 24 h, and the cellular viabilities were depicted in Figure 5. The cellular viability was higher than 90% when the concentration of CDs was below 5 mg/mL, which indicated the low cell toxicity and good biocompatibility of CDs. Furthermore, the in vitro cell uptake of CDs in Tramp C1 cells was analyzed by an inverted fluorescence microscope (Figure 6). At the 350–400 nm, 420–470 nm and 460–490 nm excitation, bright blue, green, and red luminescence in cell membrane and cytoplasma area were observed, respectively. The results indicated that the as-prepared CDs can serve as an excellent fluorescence imaging probe.

## 4. Conclusions

In this study, a facile synthetic method was used to fabricate fluorescent CDs via a hydrothermal treatment of PVP and CYS. The results showed that nitrogen and sulfur-containing groups formed on the surface of CDs, the N and S atoms were also co-doped into CDs. The CDs exhibited blue luminescence under a UV light irradiation, good water solubility, low cytotoxicity, stable photoluminescence at different pH and ionic environments, and excitation-dependent emissions. The fluorescence intensity of CDs was also found to not be affected by pH levels and stable at high ionic strength environments. The CDs also exhibited selective fluorescence quenching to Cu^2+^ with good linearity from 0.5 to 7.0 μM and a limit of detection was 0.15 μM. Furthermore, it can be also applied to the detection of Cu^2+^ in a lake water sample with satisfactory recovery. Due to their low cytotoxicity, the CDs have been used for multicolor imaging Tramp C1 cells. The results from the in vitro multicolor cellular imaging and monitoring Cu^2+^ in water samples successfully demonstrated their potential towards diverse applications in analytical areas.

## Figures and Tables

**Table 1:** Determination of cupric ions in a lake water sample (*n* = 3).

Sample	Added (μM)	Found (μM)	Recovery (%)	RSD (%)
Lake water	10.0	10.2	102.0	6.2
	50.0	45.7	91.4	3.1

## Supplementary Materials

The following are available online at https://www.mdpi.com/1420-3049/24/9/1785/s1, Figure S1: The batch-to-batch reproducibility for the synthesis CDs, Figure S2: Emission spectra of the CDs recorded with progressively longer excitation wavelengths; the values were taken in 10-nm increments. Inset: The normalized fluorescence emission spectra, Figure S3: (**a**) TEM image of the CDs, (**b**) histogram of the diameters of the CDs. Scale bar: 200 nm, Figure S4: (**a**) Normalized fluorescence intensity of the CDs at different pH levels, (**b**) normalized fluorescence intensity of the CDs at different concentrations of NaCl; (**c**) normalized fluorescence intensity of the CDs for different amounts of time during which they were irradiated by a UV lamp; (**d**) photostability of the CDs as a function of storage time (Excitation wavelength at 355 nm), Figure S5. Fluorescence responses of the CDs upon the addition of different concentrations of Cu^2+^ (0, 0.5, 1.0, 3.0, 5.0, 7.0, 10, 30, 50, 70, 100, 300, and 500 μM). The inset shows the linear correlation between (*F*_0_ *− F*)/*F*_0_ and the concentration of Cu^2+^, Table S1: Optimization of the synthetic parameters of CDs for Cu^2+^ detection, Table S2: Comparison of linear range and LODs for Cu^2+^ detection of different carbon dots-based methods.
