# Supplementary material for: Synthesis of Fluorescent Carbon Dots as Selective and Sensitive Probes for Cupric Ions and Cell Imaging
Source: Molecules. 2019 May 8;24(9):1785. doi: 10.3390/molecules24091785 (PMC6539694; doi:10.3390/molecules24091785)
Supplement: Supplementary file 1 [file molecules-24-01785-s001.pdf]

Supplementary materials

Synthesis of Fluorescent Carbon Dots as Selective and Sensitive Probes for Cupric Ions and Cell Imaging

Shu-Wei Huang[1], Yu-Feng Lin[2], Yu-Xuan Li[1], Cho-Chun Hu[1] and Tai-Chia Chiu[1,*]

[1] Department of Applied Science, National Taitung University, 95092 Taitung, Taiwan; shuwei0615@gmail.com (S.-W.H.); danny861106@gmail.com (Y.-X.L.); cchu@nttu.edu.tw (C.-C.H.)

[2] Department of Biomedical Engineering and Environmental Science, National Tsing Hua University, 30013 Hsinchu, Taiwan; assassin52852@gmail.com (Y.-F.L.)

*Correspondence: tcchiu@nttu.edu.tw; Tel.: +886-89-517-990

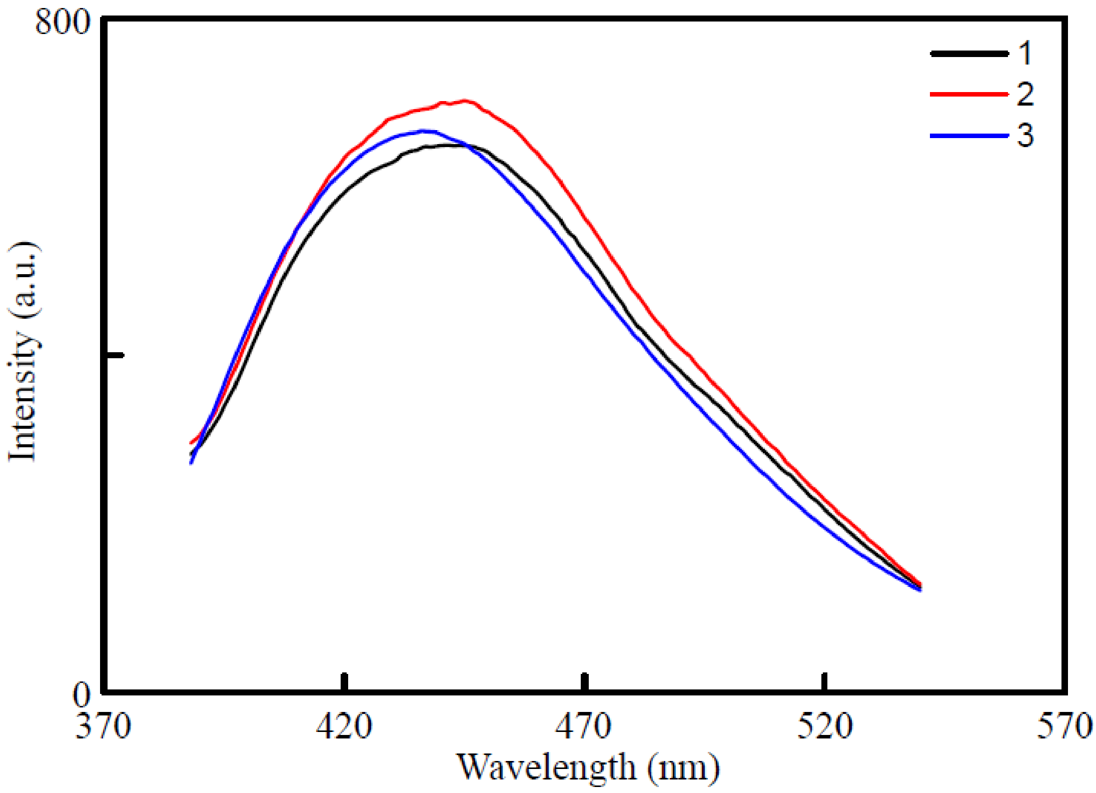

Figure S1. The batch-to-batch reproducibility for the synthesis of CDs.

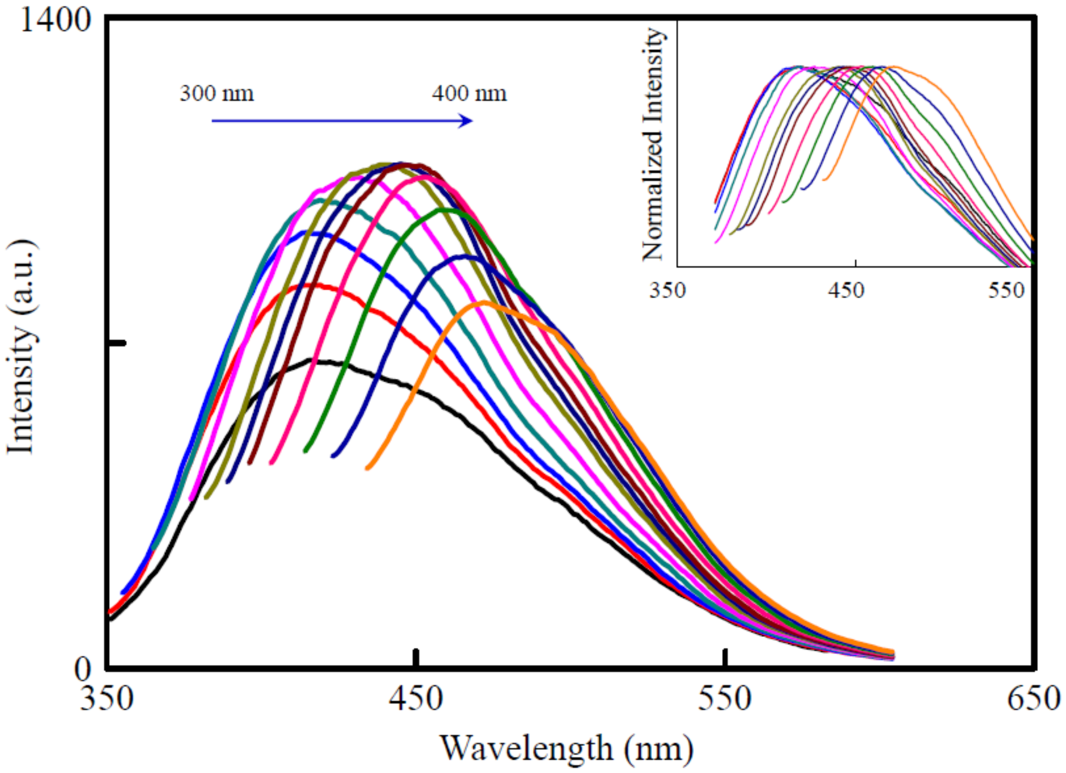

Figure S2. Emission spectra of the CDs recorded with progressively longer excitation wavelengths; the values were taken in 10-nm increments. Inset: The normalized fluorescence emission spectra.

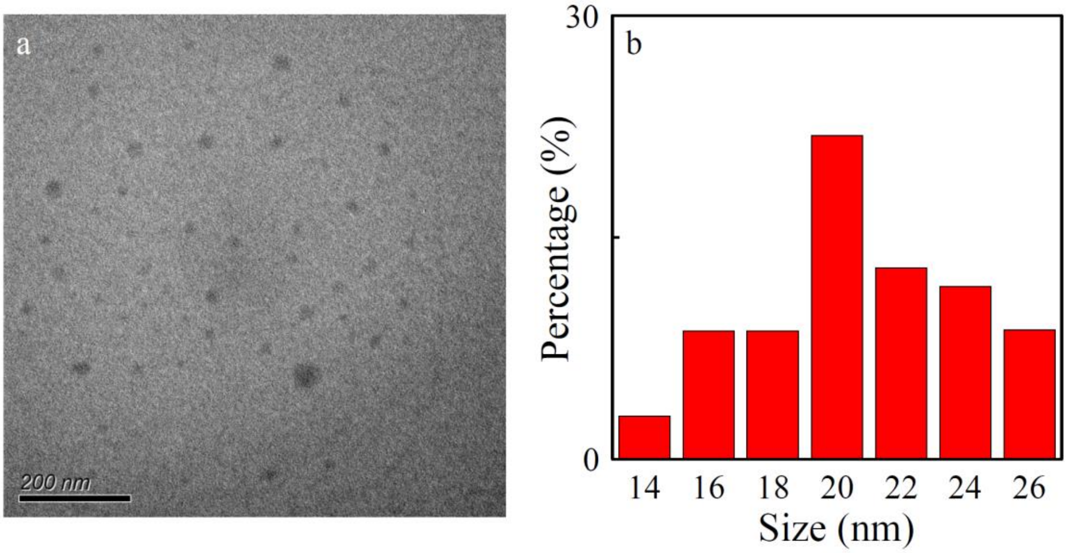

Figure S3. (**a**) TEM image of the CDs. (**b**) Histogram of the diameters of the CDs. Scale bar: 200 nm.

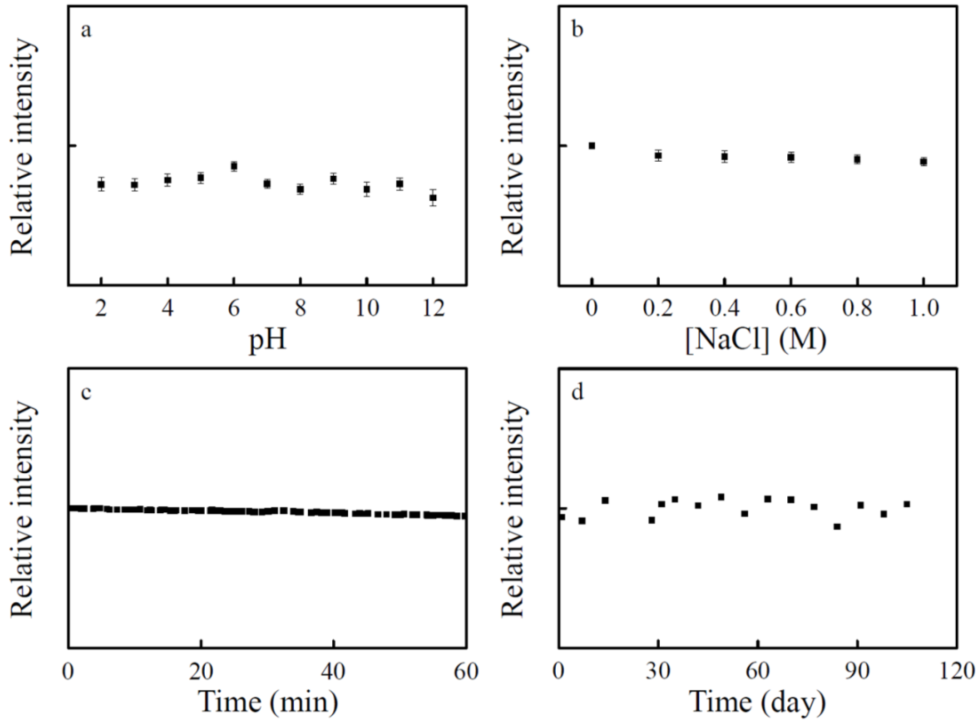

Figure S4. (**a**) Normalized fluorescence intensity of the CDs at different pH levels. (**b**) Normalized fluorescence intensity of the CDs at different concentrations of NaCl. (**c**) Normalized fluorescence intensity of the CDs for different amounts of time during which they were irradiated by a UV lamp. (**d**) Photostability of the CDs as a function of storage time (Excitation wavelength at 355 nm).

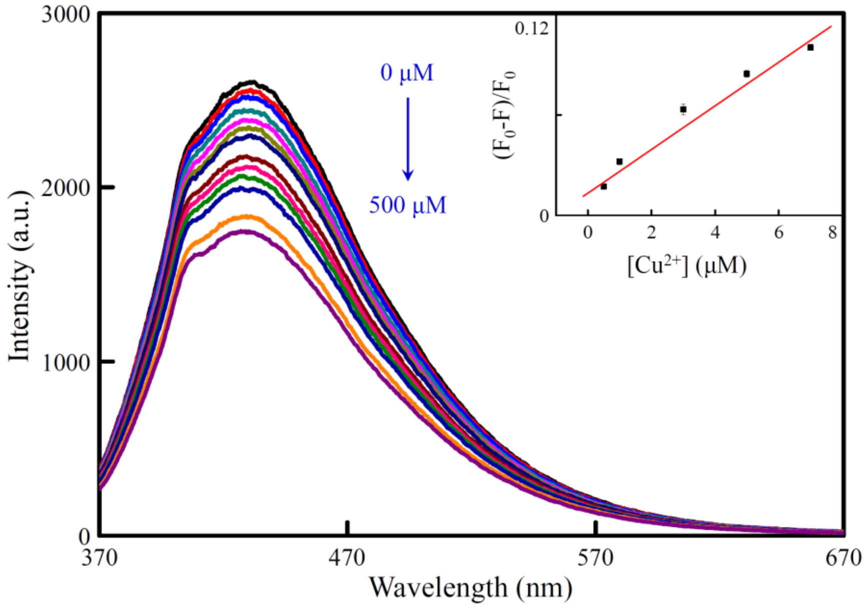

Figure S5. Fluorescence responses of the CDs upon the addition of different concentrations of Cu^{2+} (0, 0.5, 1.0, 3.0, 5.0, 7.0, 10, 30, 50, 70, 100, 300, and 500 μM). The inset shows the linear correlation between $(F_0-F)/F_0$ and the concentration of Cu^{2+}.

Table S1. Optimization of the synthetic parameters of CDs for Cu^{2+} detection.

PVP (g)	CYS (g)	Temperature (°C)	Time (h)	QY (%)	$(F_0\text{-}F)/F_0$
0.50	0.50	180	6	6.8%	0.09
0.50	0.50	180	12	7.6%	0.22
0.50	0.50	180	18	4.8%	0.05
0.50	0.50	140	12	4.7%	0.07
0.50	0.50	220	12	9.4%	0.05
0.75	0.25	180	12	9.3%	0.05
0.25	0.75	180	12	3.3%	0.03

F_0 and F are the fluorescence intensities of the probes at 455 nm in the absence and presence of the Cu^{2+} ions (10 μM), respectively.

Table S2. Comparison of linear range and LODs for Cu^{2+} detection of different carbon dots-based methods.

**Reaction Materials**	**Synthetic Approach**	**QY**	**Detection Technique**	**Linear Range (μM)**	**LODs (μM)**	**Reference**
o-Phenylenediamine, dithiothretiol	Solvothermal	~23%	Turn off	–	2	[1]
Ammonium citrate	Heating	–	Turn off	0.001–0.200	0.0004	[2]
Acacia concinna seeds	Microwave	10.20%	Turn off	0.01–10	0.0043	[3]
Sulfamide, *m*-phenylenediamine	Solvothermal	78.6%	Turn off	2–60	0.29	[4]
Lily bulbs	Microwave	17.6%	Turn off	0.05–2	0.0013	[5]
Citric acid, ethylenediamine	Hydrothermal	32.25%	Turn on	0–60	0.0031	[6]
Waste polyolefin	Ultrasonic	4.84%	Turn off	1–8	0.0006	[7]
Hexamethylenetetramine	Hydrothermal	21.7%	Turn off	0.1–40	0.09	[8]
Citric acid, L-cysteine, dextrin	Microwave	22%	Turn off	0–30	0.002	[9]
Sugarcane juice	Hydrothermal	10.7%	Turn off	5.12–100	0.76	[10]
Glucose, H_3PO_4, polyethylene glycol diamine	Heating	25%	Turn off	0.004–0.400	0.0015	[11]
Glucose, NH_3, hydrogen peroxide	Hydrothermal	32.8%	Turn off	0.1–20	0.0056	[12]
Citric acid, tris(hydroxymethyl)methyl aminomethane	Hydrothermal	62%	Turn off	0–10	0.21	[13]
Phytic acid, sodium citrate	Hydrothermal	3.5%	Turn off	0–0.0020	0.001	[14]
Citric acid, histidine	Solid-phase thermal	16%	Turn off	0.6–30	0.19	[15]
Polyvinylpyrrolidone, L-cysteine	Hydrothermal	7.6%	Turn off	0.5–7.0	0.15	This work

References:

1. Ju, B.; Zhang, T.; Li, S.; Liu, J.; Zhang, W.; Li, M.; Zhang, S.X.-A. Fingerprint identification of copper ions with absorption and emission dual-mode responses by N,S co-doped red carbon dots. *New J. Chem.* **2019**, *43*, 168-174.
2. Sun, Y.; Wei, M.; Liu, R.; Wang, H.; Li, H.; Kang, Q.; Shen, D. A smartphone-based ratiometric fluorescent device for field analysis of

soluble copper in river water using carbon quantum dots as luminophore. *Talanta* **2019**, *194*, 452-460.
3. Bhamore, J.R.; Jha, S.; Park, T.J.; Kailasa, S.K. Fluorescence sensing of Cu^{2+} ion and imaging of fungal cell by ultra-small fluorescent carbon dots derived from Acacia concinna seeds. *Sens. Actuator B-Chem.* **2018**, *277*, 47-54.
4. Dai, Y.; Liu, Z.; Bai, Y.; Chen, Z.; Qin, J.; Feng, F. A novel highly fluorescent S, N, O co-doped carbon dots for biosensing and bioimaging of copper ions in live cells. *RSC Adv.* **2018**, *8*, 42246-42252.
5. Gu, D.; Zhang, P.; Zhang, L.; Liu, H.; Pu, Z.; Shang, S. Nitrogen and phosphorus co-doped carbon dots derived from lily bulbs for copper ion sensing and cell imaging. *Opt. Mater.* **2018**, *83*, 272-278.
6. Kuang, Y.; Chen, L.; Lu, J.; Tian, X.; Yang, C.; Li, Y.; Lu, L.; Nie, Y. A carbon-dot-based dual-emission probe for ultrasensitive visual detection of copper ions. *New J. Chem.* **2018**, *42*, 19771-19778.
7. Kumari, A.; Kumar, A.; Sahu, S.K.; Kumar, S. Synthesis of green fluorescent carbon quantum dots using waste polyolefins residue for Cu^{2+} ion sensing and live cell imaging. *Sens. Actuator B-Chem.* **2018**, *254*, 197-205.
8. Liao, S.; Huang, X.; Yang, H.; Chen, X. Nitrogen-doped carbon quantum dots as a fluorescent probe to detect copper ions, glutathione, and intracellular pH. *Anal. Bioanal. Chem.* **2018**, *410*, 7701-7710.
9. Liu, Q.; Zhang, N.; Shi, H.; Ji, W.; Guo, X.; Yuan, W.; Hu, Q. One-step microwave synthesis of carbon dots for highly sensitive and selective detection of copper ions in aqueous solution. *New J. Chem.* **2018**, *42*, 3097-3101.
10. Moreira, V.A.; Toito Suarez, W.; de Oliveira Krambeck Franco, M.; Gambarra Neto, F.F. Eco-friendly synthesis of cuprizone-functionalized luminescent carbon dots and application as a sensor for the determination of copper(II) in wastewater. *Anal. Methods* **2018**, *10*, 4570-4578.
11. Omer, K.M. Highly passivated phosphorous and nitrogen co-doped carbon quantum dots and fluorometric assay for detection of copper ions. *Anal. Bioanal. Chem.* **2018**, *410*, 6331-6336.
12. Tam, T.V.; Choi, W.M. One-pot synthesis of highly fluorescent amino-functionalized graphene quantum dots for effective detection of copper ions. *Curr. Appl. Phys.* **2018**, *18*, 1255-1260.
13. Yan, F.; Bai, Z.; Chen, Y.; Zu, F.; Li, X.; Xu, J.; Chen, L. Ratiometric fluorescent detection of copper ions using coumarin-functionalized carbon dots based on FRET. *Sens. Actuator B-Chem.* **2018**, *275*, 86-94.

14. Yang, F.; He, X.; Wang, C.; Cao, Y.; Li, Y.; Yan, L.; Liu, M.; Lv, M.; Yang, Y.; Zhao, X., et al. Controllable and eco-friendly synthesis of P-riched carbon quantum dots and its application for copper (II) ion sensing. *Appl. Surf. Sci.* **2018**, *448*, 589-598.
15. Zhao, L.; Li, H.; Xu, Y.; Liu, H.; Zhou, T.; Huang, N.; Li, Y.; Ding, L. Selective detection of copper ion in complex real samples based on nitrogen-doped carbon quantum dots. *Anal. Bioanal. Chem.* **2018**, *410*, 4301-4309.
